# The *Opuntia* Effect Improves Dam-Kid Metabolic Markers, Augments Colostrum Quality and Enhances Kid-To-Dam Behavioral Interactions in Crossbred Goats and their Offspring under Semiarid-Rangeland Conditions

**DOI:** 10.3390/ani10060931

**Published:** 2020-05-28

**Authors:** Francisco G. Véliz-Deras, César A. Meza-Herrera, Sharon Herrera-Hernandez, Arnoldo Flores-Hernández, Juan M. Guillén-Muñoz, Cayetano Navarrete-Molina, Silvestre Moreno-Avalos, Rafael Rodríguez-Martínez

**Affiliations:** 1 Unidad Laguna, Universidad Autónoma Agraria Antonio Narro, Periférico Raúl López Sánchez y Carretera a Santa Fe, Torreón 27054, Coahuila, Mexico; velizderas@yahoo.com (F.G.V.-D.); sharon_13_herrera@hotmail.com (S.H.-H.); mvz_guillen@hotmail.com (J.M.G.-M.); smavalos91@hotmail.com (S.M.-A.); 2 Unidad Regional Universitaria de Zonas Áridas, Universidad Autónoma Chapingo, Bermejillo, Durango 35230, Mexico; cmeza2020@hotmail.com (C.A.M.-H.); aflores@chapingo.uruza.edu.mx (A.F.-H.); navarretemolina1977@gmail.com (C.N.-M.)

**Keywords:** goats, *Opuntia*, targeted supplementation, productive efficiency

## Abstract

**Simple Summary:**

The potential supplementation effect of protein-enriched *Opuntia* (PEO) cladodes, the flat leaf-like stem of cactus species, upon metabolic cues, colostrum, and milk quality, as well as some indicators of kid-to-dam behavioral interactions in crossbred goats under rangeland conditions, was evaluated. PEO supplementation positively influenced dam metabolic status, colostrum quality, and kid-to-dam interactions. Bio-fortified *Opuntia* cladode supplementation has emerged as an option to improve not only productive behavioral outcomes but also to enhance the sustainability of marginal rangeland goat production systems.

**Abstract:**

The possible effect of protein-enriched *Opuntia* cladode supplementation during the pre- and post-partum stages (−25 days to +15 days; day 0 = kidding) upon dam–kid metabolic status, colostrum-milk quality, and some behavioral kid-to-dam interaction in goats managed under rangeland extensive conditions was evaluated. Multiparous crossbred goats (*n* = 30), homogeneous regarding live weight (LW; 55.9 ± 1.03 kg) and body condition score (BCS; 2.5 ± 0.2 units), were randomly assigned to (1) ***protein-enriched Opuntia*** (PEO; *n* = 10; 29.8% crude protein (CP), 2.2 Mcal ME kg^−1^), (2) ***non-enriched Opuntia*** (NEO; *n* = 10; 6.4% CP, 2.1 Mcal ME kg^−1^), and (3) ***control*** (CON; *n* = 10, non-supplemented). The PEO and NEO goats were individually supplemented with *Opuntia* cladodes (250 g day^−1^; 09:00–10:00 a.m.; 25 days pre- and 15 days post-partum); then, all groups grazed in a marginal rangeland (10:00 a.m. to 06:00 p.m.). LW, BCS, and blood samples to quantify serum glucose (GLU) levels were collected weekly from day −25 up to day +15 in both dams (pre- and post-partum) and kids (post-partum). At 4 h and 8 h post-partum, kid-to-dam behavioral tests were performed; approaches (APRO, units), animal-to-animal contact (ACONT, s), latency-to-contact (LCONT, s), and high (HPB) and low (LPB) bleats were registered. The response variables LW (58.2 ± 3.5 kg), GLU from does (66.4 ± 3.3 mg/dL), colostrum fat (12.3 ± 1.15%), non-fatty solids (20.9 ± 2.1%), density (64.4 ± 7.0%), and protein (8.1 ± 0.8%), as well as milk density (31.2 ± 1.7%) and protein (3.9 ± 0.3%), favored the PEO group. Moreover, the dam-to-kid 4 h LPB (34.5 ± 4.6 frequency), as well as kid-to-dam 8 h LCONT-own (100 ± 35.5 s) and LPB (25.2 ± 6.9 frequency) also favored the PEO group. To conclude, peripartum supplementation with protein-enriched *Opuntia* cladodes emerged as a key alternative to enhance the dam–kid metabolic status, to improve colostrum quality and some milk components (density and protein), as well as to expand the kid-to-dam bond in goat production systems under marginal extensive conditions.

## 1. Introduction

In most rangelands worldwide, especially under arid and semiarid conditions, both the energy and protein consumption is found to be below the animal’s requirements because the poorest quality forages are available, especially during the dry season [1,2,3]. This situation is particularly critical during the last third of pregnancy. Certainly, the accelerated fetal growth exerts an enlarged demand for nutrients from the mother in order to maintain the availability of metabolic substrates for its own development [4]. Such an increase in the late-pregnancy requirements by fetal tissue produces a decay in the dam metabolic homeostasis, compromising the dam’s body reserves [4,5]. Nutritional deficiencies also affect placentogenesis, compromising the placental architecture and function. In turn, other processes will be affected such as differential expression of angiogenesis-involved genes, limiting cell-to-cell communication and causing a decreased level of both oxygen and nutrients. Such a physiologic scenario promotes adverse fetal outcomes because of the deleterious genetic down-regulation during fetal programming [6]. Moreover, a reduced nutritional status also compromises the normal development and function of the mammary gland [7] and colostrum quality [8]; colostrum intake supports the initiation of different anabolic processes in several tissues, encouraging postnatal body growth and organ development [9]. In turn, not only milk production and offspring development will be compromised [10], but also an increased offspring mortality rate would be expected [1,2,11]. Furthermore, such a depressed physiological scenario will also negatively affect the doe–offspring behavioral interactions and bonding while it will depress the newborn strength [5,12]. To overcome such a physiological demanding scenario is not only essential for kid survival but also to safeguard the newborn cognitive competence and recognizing ability [5,13].

Most marginal–extensive goat production schemes in arid and semi-arid ecosystems have been linked to the presence of native cacti (*Opuntia spp*) whose cladodes, the flat leaf-like stem of cactus species, have an interesting potential as alternative forage [14]. Nevertheless, the content of both fiber and crude protein (CP) is low, despite the *Opuntia* cladodes having an increased level of calcium and carbohydrates [15,16]. Interestingly, CP increases have been generated throughout a fermentation process of protein enrichment of *Opuntia* cladodes, from 12.8% (*Aspergillus niger*) [17], up to significant CP increases superior to 200% (*Saccharomyces cerevisiae*) [18]. Previous studies of our group demonstrated a positive out-of-season the effect in goats supplemented with protein-enriched *Opuntia* cladodes (i.e., the “*Opuntia effect*”), not only regarding estrus induction, estrus latency, and ovulation rate [19], but also upon corpus luteum number and diameter, as well as on embryo implantation rate [20]. Building on such ideas, our next step was to test the hypothesis that targeted supplementation of protein-enriched *Opuntia megacantha* Salm-Dyck cladodes during the last month of pregnancy and early lactation would improve the dam’s glucose concentrations, augmenting both quality and quantity of colostrum and milk, as well as the kid metabolic status, and enhancing the kid-to-dam bonding behavior in low-input, rangeland-based goat production systems under semiarid conditions.

## 2. Material and Methods

### 2.1. General

All procedures and methods used in this study regarding the use and care of animals were carried out in strict accordance with accepted international [21] and national [22] animal use and care guidelines, with institutional approval UAAAN-UL-18-4059.

### 2.2. Location, Environmental Conditions, and Goat Management

The study was conducted in a rural community in northern Mexico (25°32′ N; 103°15′ W; 1120 m) throughout the producer participatory research approach. The area has a semi-arid climate with a medium average temperature of 25.3 °C, and an average annual rainfall of 225 mm; the rainy season extends from June to October. Although the relative humidity ranges from 26.1% to 60.7%, the photoperiod ranges from 13 h 41 min (summer solstice, June) to 10 h 19 min (winter solstice, December). The vegetation type is characterized as Chihuahuan desert rangeland, which consists mainly of Creosote bush (*Larrea tridentata*), mesquite (*Prosopis glandulosa v. Glandulosa*), and lechuguilla (*Agave lechuguilla* Torr.). Goats consumed the available natural pasture and occasionally corn crop residues; they grazed daily from 10:00 a.m. to 06:00 p.m. and were penned at evening, with free access to mineral salts and water. The goatherd was managed according to the traditional procedures exerted by marginal goat producers in northern Mexico [19].

### 2.3. Animals, Experimental Groups, and Treatments

Multiparous crossbred non-pregnant goats (*n* = 30; Alpine-Saanen-Nubian × Criollo, 3–4 years old) of known fertility were induced to estrus during the natural anestrous season (i.e., April–May) with the use of intravaginal sponges and subjected to artificial insemination in order to know the approximate kidding date. The vaginal sponges contained 45 mg of fluorogestone acetate (Chronogest; Intervet International B.V., Boxmeer, The Netherlands) left in place for 10 days; 9 days after insertion of the sponges (d 3; d 0 = estrus), goats received a single intramuscular (i.m.) dose of 1 mL of prostaglandin F_2_ analog (0.075 mg of D-cloprostenol/goat; Prosolvin-C, Intervet International B.V., Boxmeer, The Netherlands). Thereafter, 26 days prior to the expected date of kidding, goats were randomly assigned into three experimental groups (*n* = 10, each) homogeneously regarding live weight (LW; 55.9 ± 1.03 kg) and body condition score (BCS; 2.5 ± 0.2 points) in a 1–4 scale (1: emaciated to 4: obese) [19,20]; the experimental groups were (1) *protein-enriched Opuntia* (PEO; 29.9% CP, 2.3 Mcal ME kg^−1^), (2) *non-enriched Opuntia* (NEO; 6.5% CP, 1.9 Mcal ME kg^−1^), and (3) control (CON; non-supplemented). PEO and NEO groups were individually supplemented in small buckets with *Opuntia* cladodes (250 g goat^−1^ day^−1^; 09:00–10:00 a.m., 25 days pre- and 15 days post-partum).

The experimental group PEO considered the protein enrichment of cladodes throughout a semisolid fermentative process previously described [19]. Briefly, small slices of *Opuntia* cladodes were mixed and inoculated with *Scharomyces cereveciae* (1%), urea (1%), and ammonium sulfate (0.1%) in a bioreactor (*NOPAFER-R*, no. 2641-IMPI, Mexico) for 10 h. Then, the enriched cladodes were semi-dried at ambient temperature for 72 h. *Opuntia* supplementation in both the NEO and PEO experimental groups was from 09:00 to 10:00 a.m. The chemical composition of both *Opuntia* treatments (NEO and PEO) is presented in Table 1. The three experimental groups were kept together during the day in the rangeland, and were separated accordingly in the evening.

### 2.4. Experimental Procedure, Measurements, and Response Variables

***Live weight, body condition, and colostrum and milk analyses.*** Although dams’ LW and BCS were recorded (day 25 pre-partum, day 0 = partum, day 7 and day 15 = post-partum), kids’ LW were recorded at day 0, day 7, and day 15. The same days, a blood sample of both the dam (day 25, day 0, day 7, and day 15) and the kids (day 0, day 7, and day 15) was collected by jugular venipuncture in order to quantify the serum glucose concentration (Accu Chek Sensor Comfort, Roche, Mexico) with a reliability of 95%. Colostrum was collected immediately after kidding; a sample of 20 mL of colostrum per goat was collected and stored at 4 °C for further chemical composition analyses (i.e., fat, protein, non-fatty solids, and density); the same procedure was performed to assess the milk chemical analysis on day 5 and day 10 post-partum (Lactycheck rapidread LC-01 RR Page and Pedersen International, Ltd., Hopkington MA, USA).

**Figure 1:**
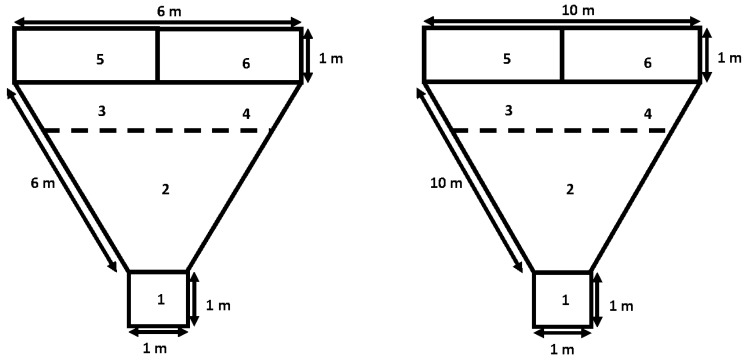
Diagram of the test arena used for the two-choice test for dams (left panel) and kids (right panel); zone 1 animals. The behavioral interaction tests considered the response variables approaches (APRO, number), animal-to-animal contact (ACONT, s), latency to contact (LCONT, s), and vocalizations with high (HPB) and low (LPB) bleats between dam-to-kid (4 h postpartum) and kid-to-dam (8 h postpartum). Data were recorded from crossbred adult female goats and their offspring managed under semiarid-subtropical rangeland conditions in Northern Mexico (25° N): starting pen, zone 2: neutral zone, zone 3 and 4: proximity zone, zone 5 and 6: holding pens for the stimulus. Note: More details were previously described in the main body of the text.

***Behavioral kid-to-dam interaction at kidding.*** Two kid-to-dam behavioral tests were carried out in the three experimental groups using the two-choice test. The dam-to-kid behavioral test was performed 4 h post-partum, evaluating the mother’s ability to recognize its own or alien kids of the same age; the behavioral test was performed in a triangular testing enclosure (Figure 1). Both the own and alien kids were placed in separate pens on the opposite side to the dam’s pen. The test started when the mother entered the isolation pen for 30 s to hear the kid’s vocalizations; then, the mother was released and the dam-to-kid interaction was recorded for 5 min. Subsequently, 8 h post-partum, the kid-to-dam behavioral test was performed using the same triangular testing enclosure, but now the ability of the kid to recognize its own or alien mother was recorded [24].

**Figure 2:**
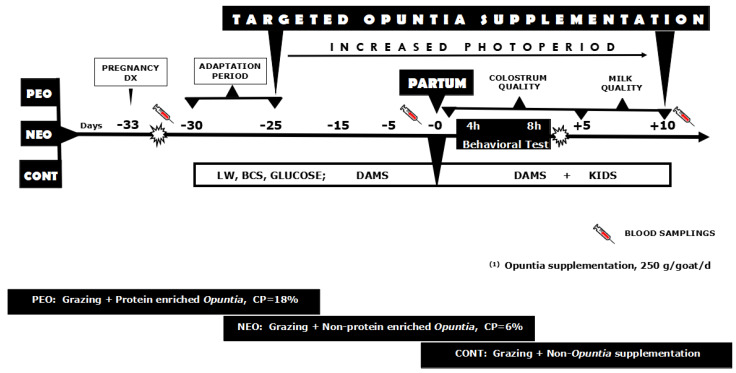
A schematic representation of the experimental protocol of targeted supplementation with *Opuntia megacantha* Salm-Dyck cladodes either protein-enriched (PEO) or non-protein enriched (NEO) and non-supplemented control (CONT) 25 days pre- and 15 days post-partum to crossbred adult female goats (*n* = 30; Alpine-Saanen-Nubian × Criollo) exposed to an increased natural photoperiod (April–June; anestrous season) under semiarid-subtropical rangeland conditions in Northern Mexico (25° N). Note: More details were previously described in the main body of the text.

The response variables considered the following kid-to-dam behavioral interactions: (1) the approaches of the test animal to their offspring or dam; (2) latency to approach, from the “neutral zone” to the “contact zone”, considering either alien or own dam/kid after exiting the start box; (3) contact between animals; and (4) the vocalization activity of dams and young (low and high bleats). The experimental protocol and the timeline of actions are shown in Figure 2.

### 2.5. Statistical Analyses

Because both dam and kids were individually fed within the treatment group, animals were considered as the experimental units. The response variables LW, BCS, GLUC, for both the dam and kids throughout the experimental period, were determined by split-plot ANOVA for repeated measures across time [25]. Serum glucose values were assessed for normality using the Shapiro–Wilk test for normality; log^10^ transformation was necessary prior to analysis to overcome skewness in the data for serum glucose. The models included the treatment effect in the main plot, which was tested using the animal within treatment as the error term. Both time and the treatment × time interaction were included in the subplot and tested by using the residual mean square (PROC MIXED). Wilcoxon rank-sum non-parametric tests (PROC NPAR1WAY) were conducted to compare the proportions of the behavioral kid-to-dam variables. Moreover, colostrum and milk composition were analyzed by PROC GLM, and significant differences detected were further investigated using the least significant difference test. Treatment was examined within sampling time when treatment × time interactions occurred. Because a treatment by time interaction occurred for the variables LW, BCS, and GLUC, simple effect least square means are presented. All statistical analyses were computed using procedures of SAS (version 9.1, Cary, NC, USA), and the significance level was set at *p* < 0.05.

## 3. Results

### 3.1. Live Weight, Body Condition, and Serum Glucose of the Dams and Kids

Regarding the dam response variables LW, BCS, and GLUC, a treatment × time interaction (*p* < 0.05) occurred (Table 2). Although the initial dam LW (i.e., 3/3 of gestation) was similar (*p* > 0.05) among groups, minor dam LW fluctuations occurred throughout the experimental period. Yet, a dam LW recovery was observed from day 0 up to day 15 post-partum (i.e., early lactation) in the PEO (+9.6%) and CONT (+1.85%) groups, with a slight decrease (−3.7%) in the NEO group, with the latter being the case despite the non-observed differences in the dam LW at parturition.

Moreover, all the groups enlarged their BCS during the early lactation stage (up to 15 days) with slight serum glucose increases in the PEO group. Regarding the kid performance, because a treatment × time interaction (*p* = 0.001) occurred for LW and GLUC, we have presented simple effects means by treatment across time (Table 3). At birth, although the kid LW was similar (*p* > 0.05) among experimental groups, the PEO kids had the largest (*p* < 0.05) serum GLUC levels (131.6 mg/dL) compared with the CONT (114.9 md/dL) and NEO (91.0 mg/dL) kids. Nonetheless, a quite interesting kid LW trend occurred throughout the experimental period. Certainly, although the kids born/raised by the PEO dams depicted a 94.2% LW increase from birth up to day 15 post-partum, minor LW increases occurred in those kids born from the NEO (36.6%) and CONT (58.3%) dams. Despite these kid LW trends being observed in the PEO kids from day 0 to day 15, such growth dynamics were not escorted by reciprocal increases in the kid serum GLUC concentrations because such values favored the NEO and CONT with regards to the PEO group, with corresponding values of −0.34%, 41.5%, and −39.4% from day 0 to day 15 postpartum.

### 3.2. Colostrum and Milk Constituents

Least square means for the response variables fat, non-fatty solids (NFS), density, and protein in colostrum and milk across treatments are presented in Table 4. Because no treatment × time interaction occurred, neither for colostrum nor for milk, response variables are presented according to nutritional supplementation groups. The largest (*p* < 0.05) observed values for the variables fat (12.3% ± 1.15%), non-fatty solids (20.9% ± 2.14%), density (64.5% ± 7.01%), and protein (8.1% ± 0.81%) of colostrum favored the PEO-supplemented goats. When comparing the observed values between the PEO vs. the CONT and NEO groups, there were observed percentage increases in the variables fat, 151.8%; NFS, 151.4%; density, 137.2%; and protein, 155.7%. With respect to the milk response variables, both density and protein favored (*p* < 0.05) PEO and NEO regarding CONT. Yet, no differences (*p* > 0.05) among treatments occurred for fat and NFS. 

Interestingly, although the colostrum average differences in the evaluated variables between the PEO vs. NEO and CONT groups ranged from 137.2% (density) to 155.7% (protein), such differences decreased with respect to milk, as such differences in the PEO and NEO vs. the CONT ranged from 114.2% (density) to 117.2% (protein).

### 3.3. Doe-To-Kid Behavior at Parturition

The response variables approaches (APRO, number), animal-to-animal contact (ACONT, s), latency to contact (LACONT, s), and vocalizations with high (HPB) and low (LPB) bleats of dam-to-kid (4 h postpartum) and kid-to-dam (8 h post-partum) interactions are shown in Table 5. Neither APRO (own: 3.0; alien: 0.4) nor ACONT (own: 169.6 s; alien: 31.1 s) or even LACONT (own: 26.0 s; alien: 66.5 s) differed among treatments for the dam-to-kid interaction, 4 h post-partum. At this time, however, an increased (*p* = 0.01) frequency in low bleats occurred in the PEO group. When evaluating the kid-to-dam behavioral interactions 8 h post-partum, no differences (*p* > 0.05) occurred among treatments with respect to APRO (own: 0.43; alien: 0.26), ACONT (own: 16.9; alien: 23.0), or pitched bleats (low: 14.0; high: 6.4). Interestingly, however, a diminished (*p* < 0.001) LACONT was observed in the PEO regarding the NEO and CONT groups.

## 4. Discussion

Our working hypothesis stated that targeted supplementation of protein-enriched *Opuntia megacantha* Salm-Dyck cladodes 25 days pre- and 15 days post-partum would improve the dam’s metabolic status, augmenting colostrum and milk quality, and enhancing not only the kid-to-dam bonding behavior but also the kid metabolic status in low-input, rangeland-based goat production systems; the results support such a hypothesis. Certainly, from 25 days pre- up to 15 days post-partum, the PEO dams depicted not only an increased LW but also a positive serum GLUC status. In addition, despite no differences in the kid LW at birth occurring among treatments, from day 0 up to day 15, the PEO kids showed an enhanced LW, although non-escorted by increases in the kid GLUC levels. Moreover, not only the colostrum and milk quality but the behavioral dam-to-kid 4 h post-partum interactions revealed an increased low bleat frequency (i.e., *quiet vocalizations produced with the mouth closed*), whereas reduced latency-to-contact was found in the kid-to-dam 8 h post-partum, all of them favoring to the PEO group. These results are consistent with the outcomes obtained with a supplementation (0.500 kg of corn) offered from 14 days pre-partum [12]. Our results are also in accordance with previous results, which stated that increases in live weight and metabolic status [24,26,27] or in milk quality [28] were observed in energy protein-supplemented goats under grazing-marginal conditions. These outcomes emphasize the benefits to offer nutritional peri-partum supplementation in range-based goat production systems, generating positive effects upon both does and kids. Moreover, such a physiologic scenario suggests a PEO dynamic effect of supplementation [29,30] from day –25 to day 0 upon the dam’s LW; BCS; serum GLUC; colostrum quality; and, to a lesser degree, on milk quality. Our results also suggest a PEO supplementation “acute effect” upon the behavioral interactions, not only dam-to-kid at 4 h post-partum, but also kid-to-dam at 8h post-partum [2,4]. Interestingly, the possible dynamic and acute effects exerted by the PEO supplementation occurred despite the relatively reduced BCS observed in the dams at kidding (2.23 ± 0.34 units), irrespective of the treatment group [12]. The obtained results for the kid’s LW at day 0 and day 15 agree with previous reports [24,26,27].

With a global goat milk production of 18.7 M tons—almost 2% of the worldwide milk production—goat milk is of particular importance in Asia, Africa, and the Mediterranean region, with the largest goat milk volumes produced by Asia (57%) and Africa (25%), followed by Europe (14%) and the Americas (4%) [31]. The colostrum quality in our study revealed that the largest (*p* < 0.05) values for fat, non-fatty solids, density, and protein favored to the PEO-supplemented goats. These results are coherent with the positive effect of a 12 day pre-partum energy supplementation upon colostrum quality [12]. The observed results in our study also suggest that protein supplementation may potentially have affected udder development and function, and in turn, colostrum quality [7] and milk quality [28].

The enhanced level of protein supply in the PEO group may have served as a source for an enhanced synthesis of immunoglobulins, augmenting, in turn, the total protein content in the colostrum. Studies in sheep have demonstrated a decreased immunoglobulin level in malnourished ewes [32]. Moreover, the observed higher content of fat and non-fatty solids probably resulted from a greater passage and synthesis of nutrients to the mammary gland; certainly, an inadequate nutrition level from middle to late gestation reduces colostrum quality and quantity [33]. Colostrum becomes the first source of antibodies before the immune system is fully functional and its consumption is a primary factor for the survival of the newborn [9]. The last is of key importance to enhance the sustainability of any goat production system, especially under marginal-range conditions [1].

In our study, the milk density and protein level favored the PEO-supplemented group, opposite to previous reports stating that no beneficial effects on composition of milk occurred in pre-partum supplemented goats [34,35]. However, our study is in agreement with supplementation of 18% CP/kg DM from day 35 pre- up day 7 post-partum upon the milk protein content [24]. The similar milk fat percentage observed among treatment groups in our study differs from a previous report from our group where nutritional supplementation promoted increases in milk components, especially fat (4.56% ± 0.18%) and lactose (5.07% ± 0.08%) under similar rangeland conditions [28]. Therefore, it can be suggested that the PEO supplementation may have not promoted a lipo-mobilization effect upon the plasma concentration of non-esterified fatty acids, which are required for the synthesis of milk fat in the mammary gland [36]. Moreover, the fact that the *Opuntia* supplement mainly affected the colostrum components instead of the milk composition could have occurred because colostrogenesis begins around 7 days pre-partum, and thus the PEO supplementation may have positively affected the dam’s metabolic status. Certainly, because the PEO supply was offered 25 days pre-partum, it could be assumed as a nutritional supplementation effect throughout the so-called “dynamic effect” [4].

As reviewed, increased offspring mortality reduces the sustainability of marginal, range-based goat production systems [2]; dam pre-kidding metabolic status affects not only the kid’s birth weight [37] but decreases udder development, and colostrum and milk quality as well [7,9,34], compromising maternal behavior and the dam-to-kid bonding [38]. With respect to the dam-to-offspring interactions evaluated at 4 h post-partum, an increased low bleats frequency occurred in the PEO group. Moreover, when evaluating the kid-to-dam behavior 8 h post-partum, a reduced latency-to-contact was observed in the PEO regarding the NEO and CONT groups. These results suggest that supplementation in the last-third of gestation in goats under marginal grazing conditions promotes an enhanced maternal response while augmenting the offspring ability to recognize their mothers. Although such results differ with previous studies [13,24,39], our study agrees with and supports previous findings from Luna-Orozco et al. [24], which suggests that kids are able to identify their own mother against the alien dam during the first 8 h, a conclusion also supported by Poindron et al. [40]. It has been proposed that the offspring from mothers facing pregnancy dietary restrictions are not impaired in their ability to discriminate against their mother [13]. In our study, the latency-to-contact to a kid’s own mother was reduced in the PEO group, suggesting that supplementation enhanced the kid’s recognition ability, possibly generated because of a greater supply of nutrients through two maternal supporting strategies: an increased placental function throughout the last weeks of pregnancy, as well as an increased colostrum quality for the PEO goats. Nonetheless, a previous study stated that the offspring of the supplemented dams showed favorable differences in their behavior, yet independently of the kid’s birth weight [12].

## 5. Conclusions

To conclude, targeted supplementation with protein-enriched *Opuntia megacantha* Salm-Dyck cladodes (PEO; 25 days pre- and 15 days post-partum) improved the dam’s metabolic status, augmented colostrum quality and some milk components, and enhanced not only the kid-to-dam bonding behavior but the kid metabolic status (i.e., serum glucose) at birth in low-input, rangeland-based goat production systems. Certainly, the PEO dams depicted not only an increased LW but also a positive serum glucose status. Moreover, despite the non-treatment differences in the kid LW at birth, the PEO kids depicted an enhanced LW from day 0 up to day 15, although not followed by increases in the serum kid GLUC concentrations. Moreover, the behavioral interactions dam-to-kid 4 h post-partum revealed an increased low bleat frequency, whereas it reduced kid-to-dam latency-to-contact at 8 h post-partum, accompanied by improved colostrum quality. The interesting multidimensional responses observed in the PEO-supplemented group generated remarkable physiological plasticity and compensatory responses that could potentially promoted physiological and behavioral scenarios prone to enhanced dam and kid productive outcomes. Such responses are required to improve the sustainability in rangeland-marginal goat production systems.

## Figures and Tables

**Table 1:** Mean chemical composition (SD), dry-basis, of *Opuntia megacantha* Salm-Dyck cladodes, either protein-enriched (PEO) or non-protein enriched (NO), and offered as supplement to adult mix-breed (Alpine-Saanen-Nubian × Criollo; *n* = 30) female goats exposed to an increased natural photoperiod (April–June; anestrous season) under semiarid-subtropical rangeland conditions in Northern Mexico (25° N).

Fraction	NEO, Fresh	NEO, Dry	PEO, Fresh	PEO, Dry
DM, %	12.1	91.7	11.5	92.7
CP, %	6.5	4.8	29.9	20.8
NDF, %	21.8	14.7	18.1	17.2
ADF, %	19.3	11.9	16.3	17.4
NFC, %	43.2	53.3	24.1	33.7
TDN, %	53.4	61.0	57.8	56.8
NEm, Mcal/kg DM	1.9	2.3	2.3	2.2
Ash, %	27.5	24.3	25.6	26.4

DM: Dry matter; CP: Crude Protein; NDF: Neutral Detergent Fiber; ADF: Acid Detergent Fiber; NFC: Non-Fiber Carbohydrate; TND: Total Digestible Nutrients; NEm: Net Energy for maintenance. NEm was calculated using equations proposed by the NRC [23].

**Table 2:** Least square means for live weight (kg) body condition score (units) and serum glucose concentrations (mg/dL) across time in crossbreed (Alpine-Saanen-Nubian × Criollo; *n* = 30) dams supplemented with *Opuntia megacantha* Salm-Dyck cladodes, either natural (NEO) or protein-enriched (PEO) or non-supplemented control (CONT), exposed to an increased natural photoperiod (April–June; anestrous season) under semiarid-subtropical rangeland conditions in Northern Mexico (25° N).

Time					
Treatments	−25	0 (Partum)	+7	+15	S.E. ^1^
Dam Live Weight, kg; Trt × Time, *p* = 0.004					
CONT	61.2 ^a^	54.2 ^ab^	54.4 ^ab^	55.3 ^ab^	3.3
NEO	60.7 ^a^	54.3 ^ab^	53.4 ^b^	52.5 ^b^	3.7
PEO	58.2 ^a^	53.1 ^b^	54.8 ^ab^	58.2 ^ab^	3.5
Dam Body Condition Score, units; Trt × Time, *p* = 0.003					
CONT	2.7 ^ab^	2.2 ^d^	2.5 ^abc^	2.7 ^ab^	0.32
NEO	2.8 ^a^	2.3 ^cd^	2.7 ^ab^	2.5 ^bcd^	0.33
PEO	2.7 ^ab^	2.2 ^d^	2.3 ^cd^	2.7 ^ab^	0.37
Dam Glucose, mg/dL; Trt × Time, *p* = 0.004					
CONT	60.0 ^ab^	55.3 ^ab^	49.7 ^b^	53.7 ^b^	3.2
NEO	58.1 ^ab^	51.7 ^b^	50.8 ^b^	48.1 ^b^	3.6
PEO	57.4 ^ab^	66.4 ^a^	57.4 ^ab^	54.0 ^b^	3.3

^a–d^ Least-square-means without a common superscript across time are different (*p* < 0.05). ^1^ Most conservative standard error is presented.

**Table 3:** Least square means for live weight (kg) and serum glucose concentrations (mg/dL) across time in kids born from adult crossbreed (Alpine-Saanen-Nubian × Criollo; *n* = 30) female goats supplemented with *Opuntia megacantha* Salm-Dyck cladodes, either natural (NEO) or protein-enriched (PEO) or non-supplemented control (CONT), exposed to an increased natural photoperiod (April–June; anestrous season) under semiarid-subtropical rangeland conditions in Northern Mexico (25° N).

Time				
Treatments	0 (Partum)	+7	+15	S.E. ^1^
Kid Live Weight, kg; Trt × Time, *p* = 0.004				
CONT	3.6 ^de^	4.5 ^cd^	5.7 ^b^	0.289
NEO	4.1 ^cde^	4.6 ^c^	5.6 ^b^	0.278
PEO	3.5 ^e^	4.3 ^cde^	6.8 ^a^	0.276
Kid Glucose, mg/dL; Trt × Time, *p* = 0.004				
CONT	114.9 ^ab^	98.8 ^bc^	114.5 ^ab^	7.59
NEO	91.0 ^bc^	108.3 ^abc^	128.8 ^a^	7.52
PEO	131.6 ^a^	87.8 ^bc^	79.8 ^c^	7.47

^a–e^ Least-square means without a common superscript among treatments across time are different (*p* < 0.05). ^1^ Most conservative standard error is presented.

**Table 4:** Least square means for the response variables fat (%), non-fatty solids (NF solids, %), density (%), and protein (%) in colostrum and milk from adult crossbreed (Alpine-Saanen-Nubian × Criollo; *n* = 30) female goats supplemented with *Opuntia megacantha* Salm-Dyck cladodes, either natural (NEO) or protein-enriched (PEO) or non-supplemented control (CONT), exposed to an increased natural photoperiod (April–June; anestrous season) under semiarid-subtropical rangeland conditions in Northern Mexico (25° N).

Treatments	Fat %	NF Solids %	Density %	Protein %
Colostrum (*p* < 0.05)				
CONT	8.9 ^ab^	14.3 ^b^	48.7 ^b^	5.4 ^b^
NEO	7.3 ^b^	13.3 ^b^	45.3 ^b^	5.0 ^b^
PEO	12.3 ^a^	20.9 ^a^	64.5 ^a^	8.1 ^a^
S.E.^1^	1.15	2.14	7.01	0.81
Milk (*p* < 0.05)				
CONT	7.4 ^a^	9.1 ^a^	27.5 ^b^	3.2 ^b^
NEO	6.1 ^a^	9.6 ^a^	31.6 ^a^	3.6 ^ab^
PEO	6.4 ^a^	9.9 ^a^	31.2 ^a^	3.9 ^a^
S.E. ^1^	0.82	0.38	1.71	0.30

^a,b^ LS-means without a common superscript across time are different (*p* < 0.05). ^1^ Most conservative standard error is presented.

**Table 5:** Least square means for behavioral responses including approaches (APRO, number), animal-to-animal contact (ACONT, s), latency to contact (LACONT, s), and vocalizations with high (HPB) and low (LPB) bleats of dam-to-kid (4 h postpartum) and kid-to-dam (8 h postpartum). Kids born from adult crossbreed (Alpine-Saanen-Nubian × Criollo; *n* = 30) female goats supplemented with *Opuntia megacantha* Salm-Dyck cladodes, either natural (NEO) or protein-enriched (PEO) or non-supplemented control (CONT), exposed to an increased natural photoperiod (April–June; anestrous season) under semiarid-subtropical rangeland conditions in Northern Mexico (25° N).

	APRO ^1^		ACONT, s ^2^		LACONT, s ^3^		BLEATS ^4^	
Treatments	Own	Alien	Own	Alien	Own	Alien	Low	High
Dam-to-kid, 4 h postpartum								
CONT	4.4 ^a^	0.8 ^a^	130.4 ^a^	27.8 ^a^	8.2 ^a^	29.7 ^a^	28.4 ^a^	0.5 ^a^
NEO	2.1 ^a^	0.0 ^a^	240.2 ^a^	16.9 ^a^	31.5 ^a^	25.0 ^a^	4.2 ^b^	0.1 ^a^
PEO	2.7 ^a^	0.5 ^a^	139.1 ^a^	48.6 ^a^	38.5 ^a^	14.5 ^a^	34.5 ^a^	1.4 ^a^
S.E. ^5^	1.03	0.18	36.8	16.2	16.4	20.8	4.6	0.5
*p*-value	0.29	0.12	0.61	0.51	0.12	0.68	0.001	0.48
Kid-to-dam, 8 h postpartum								
CONT	0.0 ^a^	0.0 ^a^	0.0 ^a^	0.0 ^a^	174.7 ^a^	271.0 ^a^	4.2 ^b^	3.0 ^a^
NEO	0.5 ^a^	0.4 ^a^	5.6 ^a^	28.0 ^a^	248.4 ^a^	252.6 ^a^	12.7 ^b^	10.8 ^a^
PEO	0.8 ^a^	0.4 ^a^	45.2 ^a^	41.8 ^a^	100.1 ^b^	242.7 ^a^	25.2 ^a^	5.4 ^a^
S.E. ^5^	0.03	0.2	15.4	20.1	35.5	34.0	6.9	4.9
*p*-value	0.42	0.76	0.22	0.48	0.03	0.87	0.06	0.52

^1^ Approaches is the number of approaches of the test animal to either the offspring or the dam. ^2^ Contact between animals is the time spent by the animals seeking the stimulus animal. ^3^ Latency to contact is the time taken to make contact with either the own or alien animal at the end of the test area. ^4^ Low bleats refer to the quiet vocalizations produced with the mouth closed. High bleats refer to the loud calls produced with the mouth opened. ^5^ Most conservative standard error is presented. ^a,b^ Means without a common superscript within column-variable-time are different (*p* < 0.05).
